# Nrf2 Deficiency Exaggerates Doxorubicin-Induced Cardiotoxicity and Cardiac Dysfunction

**DOI:** 10.1155/2014/748524

**Published:** 2014-05-06

**Authors:** Siying Li, Wenjuan Wang, Ting Niu, Hui Wang, Bin Li, Lei Shao, Yimu Lai, Huanjie Li, Joseph S. Janicki, Xing Li Wang, Dongqi Tang, Taixing Cui

**Affiliations:** ^1^Shandong University Qilu Hospital Research Center for Cell Therapy, Key Laboratory of Cardiovascular Remodeling and Function Research, Qilu Hospital of Shandong University, Jinan 250012, China; ^2^Department of Cell Biology and Anatomy, University of South Carolina, School of Medicine, Columbia, SC 29208, USA

## Abstract

The anticancer therapy of doxorubicin (Dox) has been limited by its acute and chronic cardiotoxicity. In addition to a causative role of oxidative stress, autophagy appears to play an important role in the regulation of Dox-induced cardiotoxicity. However, the underlying mechanisms remain unclear. Accordingly, we explored a role of nuclear factor erythroid-2 related factor 2 (Nrf2) in Dox-induced cardiomyopathy with a focus on myocardial oxidative stress and autophagic activity. In wild type (WT) mice, a single intraperitoneal injection of 25 mg/kg Dox rapidly induced cardiomyocyte necrosis and cardiac dysfunction, which were associated with oxidative stress, impaired autophagy, and accumulated polyubiquitinated protein aggregates. However, these Dox-induced adverse effects were exaggerated in Nrf2 knockout (Nrf2^−/−^) mice. In cultured cardiomyocytes, overexpression of Nrf2 increased the steady levels of LC3-II, ameliorated Dox-induced impairment of autophagic flux and accumulation of ubiquitinated protein aggregates, and suppressed Dox-induced cytotoxicity, whereas knockdown of Nrf2 exerted opposite effects. Moreover, the exaggerated adverse effects in Dox-intoxicated Nrf2 depleted cardiomyocytes were dramatically attenuated by forced activation of autophagy via overexpression of autophagy related gene 5 (Atg5). Thus, these results suggest that Nrf2 is likely an endogenous suppressor of Dox-induced cardiotoxicity by controlling both oxidative stress and autophagy in the heart.

## 1. Introduction

The anticancer drug doxorubicin (Dox) (also referred to as adriamycin) is highly effective in the treatment of a broad range of cancers; however, it is associated with dose-dependent acute and chronic cardiotoxicity, which significantly limits its chemotherapeutic dosage [1–4]. The cause of Dox-induced cardiotoxicity is multifactorial and includes free radical-induced mitochondrial damage, DNA damage, inhibition of DNA and protein synthesis, and myofiber degeneration, which cumulatively leads to myocardial apoptotic and/or necrotic cell death. Nevertheless, the precise pathophysiology of Dox-induced cardiotoxicity is not fully understood.

That oxidative stress is the primary cause of Dox-induced cardiomyopathy has been the prevailing hypothesis [3, 4]. However, antioxidant approaches of nonselective reactive oxygen species (ROS) scavenging for the treatment of Dox-induced cardiomyopathy have been shown to be ineffective. Therefore efficacious therapy may require more specific targeting of either the source of oxidative stress or the endogenous antioxidant defense system. However, such specific targets remain to be identified.

Nuclear factor erythroid-2 related factor 2 (Nrf2) is a master transcription factor in controlling the basal and inducible expression of a battery of antioxidant genes and other cytoprotective phase II detoxifying enzymes [5]. We have demonstrated that Nrf2 is a negative regulator of cardiac pathological remodeling and dysfunction via suppressing oxidative stress in diverse pathological settings [6, 7]. While it has been documented that Nrf2 plays a mediator role in hydrogen sulfide-mediated suppression of oxidative stress-induced cardiac dysfunction [8], we and others have demonstrated that Nrf2 might be a drug target for the treatment of cardiomyocyte injury and cardiac dysfunction [9, 10]. Of interest, a recent report has revealed that Nrf2 is able to enhance autophagic clearance of toxic ubiquitinated protein aggregates secondary to ROS formation, suggesting a novel mediator role of Nrf2 for sufficient activation of autophagy [11]. Considering the causative role of oxidative stress and the protective effect of sufficient autophagy activation in Dox-induced cardiotoxicity as well as the Nrf2-mediated antioxidant defense and sufficient activation of autophagy, it is conceivable that Nrf2 is a negative regulator of Dox-induced cardiomyopathy.

Therefore, in the present study, we explored the role of Nrf2 in the regulation of Dox-induced cardiomyopathy with a focus on oxidative stress and autophagic activity in the heart. We demonstrate that loss of Nrf2 function exaggerates Dox-induced oxidative stress, insufficient autophagic activities, and cardiomyocyte necrosis, as well as cardiac dysfunction. These results indicate that Nrf2 acts as a critical negative regulator of Dox-induced cardiomyopathy, thereby identifying a potential, novel target for the treatment of Dox-induced cardiomyopathy.

## 2. Results

### 2.1. Dox-Induced Cardiac Oxidative Stress, Toxicity, and Dysfunction

**Figure 1:**
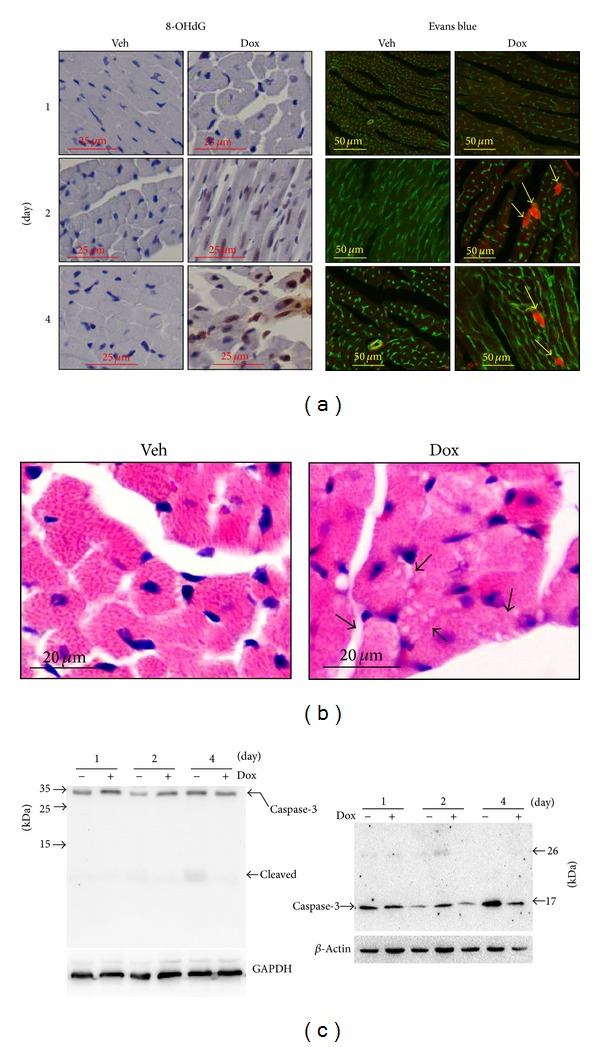
Doxorubicin- (Dox-) induced oxidative stress and necrosis in the heart. Male 10–12 wk old WT mice (C57BL/6J) were subjected to single intraperitoneal injection of 25 mg/kg Dox or vehicle (Veh) control, and then the hearts (*n* = 4 for each group at indicated time point) were harvested for immunochemical staining and Western blot analysis. (a) Representative images of 8-OHdG and Evans blue labelling. Left panel: brown staining indicates 8-OHdG. Right panel: green indicates cardiomyocyte membrane. Red indicates necrotic myocardium. (b) Representative HE staining of left ventricle tissue sections from the mice 4 days after treatment. (c) Representative immunoblots of caspase-3 and cleaved caspase-3.

Considering oxidative stress to be the primary cause of Dox-induced cardiomyopathy [3, 4], we first determined myocardial oxidative stress and cell death in the acute model of Dox-induced cardiotoxicity which has been characterized as a rapid cardiomyocyte necrosis and reduced cardiac output within 4 days after a single treatment of Dox [12, 13]. Of note, a single intraperitoneal injection of Dox (20 mg/kg) results in reduction of left ventricle fraction shorting (FS%) in C57BL/6J mice [13]. Following a single intraperitoneal injection of Dox (25 mg/kg), there was a time dependent onset of oxidative stress in the heart, which appeared at day 1 and was sustained by day 4 after injection (Figure 1(a)). There was also substantial cardiomyocyte necrosis in the heart as evidenced by cytoplasmic vacuolization and sporadic Evans blue dye uptake in cardiomyocytes [12, 14] as well as the absence of increases in myocardial cleaved 12 kDa and 17 kDa caspase 3 (Figure 1(c)), a hallmark of apoptosis [15]. Moreover, Dox treatment for 4 days led to decreases in systolic left ventricle posterior wall thickness (LVPW; s), LV volumes during both systole and diastole, and FS% (Table 1). These results reveal that in the acute model of Dox-induced cardiomyopathy a rapid onset of myocardial oxidative stress is associated with cardiomyocyte necrosis, loss of myocardial mass, and cardiac dysfunction.

### 2.2. Dox-Induced Impairment of Autophagy and Accumulation of Ubiquitinated Proteins in the Heart

Macroautophagy (commonly known as autophagy) is an evolutionarily conserved process that mediates the lysosome-dependent turnover of macromolecules and entire organelles [16]. The autophagy begins with formation of the autophagosome, a double-membrane structure of unknown origin that engulfs cytoplasmic contents, and then fuses it with a lysosome to form autolysosome (known as autophagosome mature) which then leads to proteolysis of engulfed materials. Importantly, autophagy and the ubiquitin proteasome system (UPS) are the two major routes for the complete degradation/clearance of abnormal protein products within cells [17, 18]. UPS is usually effective in clearing soluble misfolded or damaged proteins via ubiquitination of the target proteins whereas autophagy is generally efficient in clearing less soluble or insoluble ubiquitinated protein aggregates which are usually toxic to cells. Therefore, autophagy plays a crucial role in the maintenance of cellular homeostasis and the cellular adaptive protection against diverse stress.

**Figure 2:**
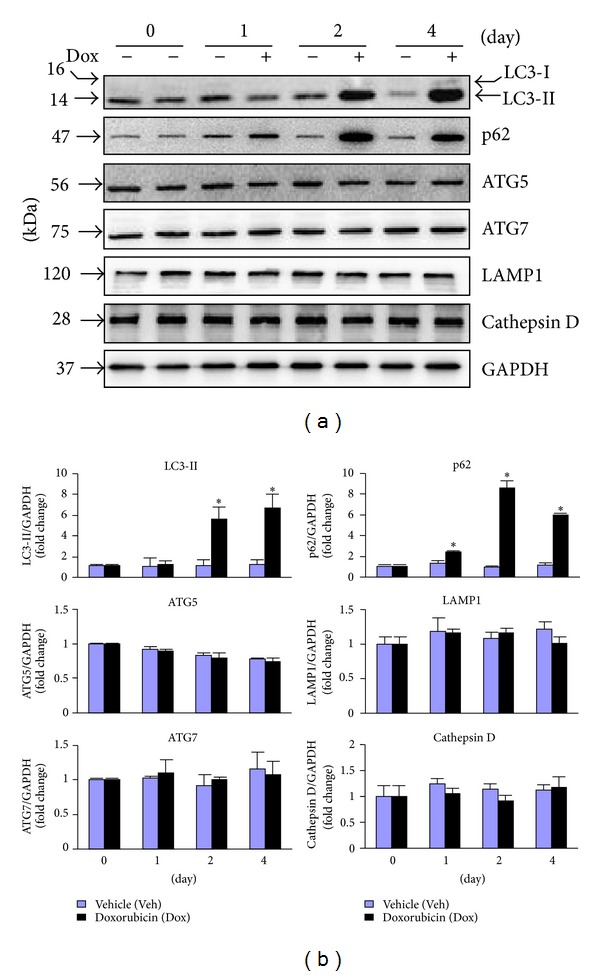
Dox-induced autophagic activity in the heart. Male 10–12 wk old WT mice (C57BL/6J) were treated as in Figure 1, and then hearts (*n* = 4 for each group at indicated time point) were harvested for Western blot analysis. (a) Representatives of immunoblots as indicated. (b) Semiquantified analysis of protein expression as indicated (*n* = 4). *P < 0.05 versus Veh in the same experimental group.

**Figure 3:**
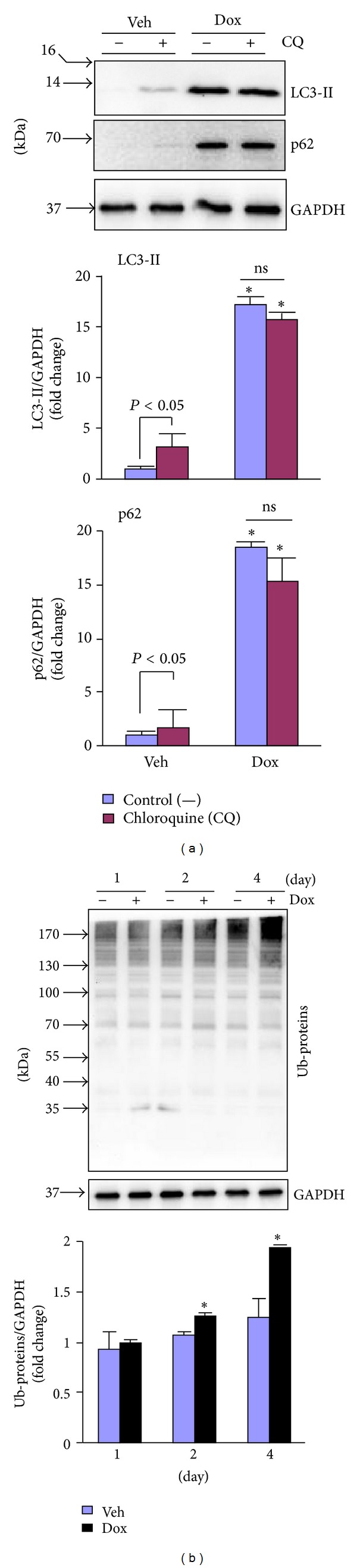
Dox-induced impairment of autophagy in the heart. (a) Male 10–12 wk old WT mice (C57BL/6J) were treated with Dox as in Figure 1 for 4 days and then subjected to measurement of autophagic flux in the heart (*n* = 4 for each group). Upper panel: representative immunoblots as indicated. Lower panel: semiquantified analysis of LC3-II and p62 protein expression. *P < 0.05 versus Veh. (b) LV lysates as in Figure 2 were subjected to Western blot analysis of polyubiquitinated proteins (Ub-proteins). Upper panel: representative immunoblots as indicated. Lower panel: semiquantified analysis of Ub-proteins level. *P < 0.05 versus Veh in the same experimental group.

It has been established that during the autophagic response, microtubule-associated protein 1 light chain 3 (LC3)-I, a 16-kDa homologue of Atg8 in yeast, is processed and lipid conjugated, resulting in LC3-II, a 14-kDa active isoform that migrates from the cytoplasm to isolation membranes and autophagosomes [16]. The protein abundance of LC3-II usually reflects the steady level of autophagosomes, which is dependent on a balance between autophagosome synthesis and autophagosome clearance via lysosomes [16]. p62 is an adaptor protein for autophagosome clearance via its fusion with lysosome and correlates inversely with autophagic activity [19]. Therefore, the impact of Dox treatment on myocardial autophagic activity was evaluated by determining expression of LC3-I and -II, p62, autophagy related gene (Atg)5 and Atg7 which are critical for autophagosome formation [16], lysosomal membrane sialoglycoprotein (LAMP)1 and lysosomal protease, and cathepsin D. We found that Dox led to time-dependent increases in the protein expression of LC3-I and II and the accumulation of p62; however, it minimally regulated the protein expression of Atg5, Atg7, LAMP, and cathepsin D (Figure 2), indicating impaired autophagic activity. To examine whether the accumulated LC3-II and p62 proteins were due to impaired autophagosome degradation, we measured myocardial autophagic flux, a more reliable measurement of autophagic activity [16], in the mice treated with vehicle and Dox. Treatment of chloroquine (CQ), an inhibitor of autophagosome fusion with lysosome, led to increased myocardial protein levels of LC3-II and p62 (autophagic flux) in control vehicle treated mice (Figure 3(a)), demonstrating a sufficient autophagic activity in the normal heart. Although Dox treatment resulted in increased myocardial protein levels of LC3-II and p62, CQ treatment had no impact on the myocardial protein expression of LC3-II and p62 in the mice treated with Dox (Figure 3(a)), suggesting an impaired autophagic flux in the Dox-intoxicated heart. In addition, Dox treatment resulted in time-dependent accumulation of ubiquitinated proteins in the heart (Figure 3(b)). Taken together, these results indicate that Dox treatment resulted in impaired autophagic activity and accumulated ubiquitinated proteins in the heart.

### 2.3. Dox-Induced Nrf2 Activation in the Heart

**Figure 4:**
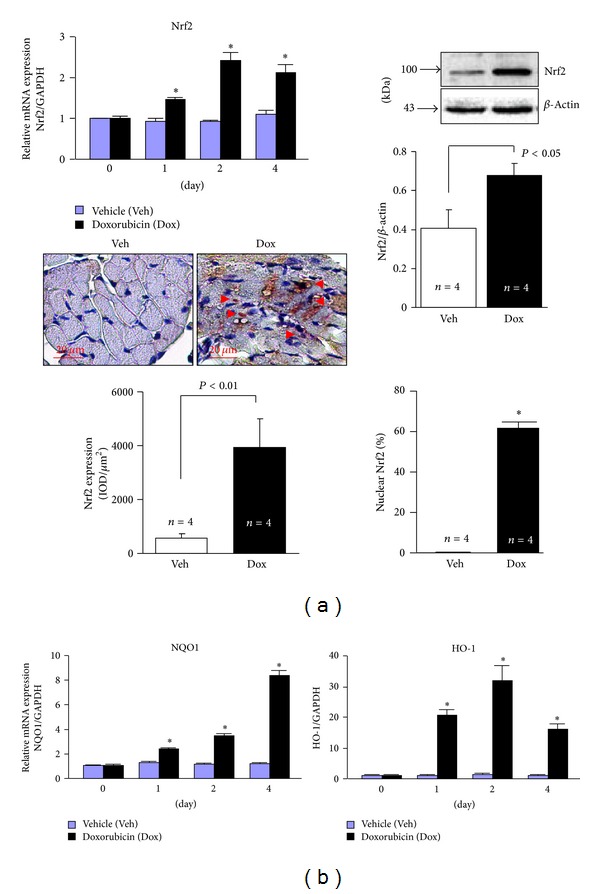
Dox-induced activation of Nrf2 in the heart. Male 10–12 wk old WT mice (C57BL/6J) were treated with Dox as in Figure 1 and the hearts (*n* = 4 for each group at indicated time point) were harvested for Q-PCR analysis, Western blot analysis, and immunochemical staining. (a) Left upper panel: Q-PCR analysis of Nrf2 mRNA expression. *n* = 4, *P < 0.05 versus Veh in the same experimental group. Right upper panel: Western blot analysis of Nrf2 protein expression in the heart from mice 4 days after treatment. Lower panel: immunochemical staining of Nrf2 protein in a heart of mouse 4 days after treatment. *P < 0.05 versus Veh. (b) Q-PCR analysis of NQO1 and HO-1 mRNA expression. *n* = 4, *P < 0.05 versus Veh in the same experimental group.

Previously, we have demonstrated that Nrf2 is a critical endogenous inhibitor of cardiac maladaptive remodeling and dysfunction via suppressing oxidative stress in the heart [6]. Of note, a recent report has revealed that Nrf2 is able to enhance autophagic clearance of toxic ubiquitinated protein aggregates secondary to ROS formation, suggesting a novel mediator role of Nrf2 for sufficient activation of autophagy [11]. Therefore, these results suggest that Nrf2 may be capable of protecting against Dox-induced cardiomyopathy via its abilities to control oxidative stress and autophagic activity. To explore the potential role of Nrf2 in Dox-induced cardiomyopathy, we then examined the Nrf2 signaling in the heart after Dox treatment. As shown in Figure 4(a), Dox treatment enhanced myocardial Nrf2 mRNA levels in a time dependent manner. Dox treatment also enhanced myocardial Nrf2 protein levels and triggered Nrf2 nuclear translocation in cardiomyocytes 4 days after the treatment (Figure 4(a)). In addition, Dox treatment led to increases in myocardial mRNA levels of NAD(P)H:quinone oxidoreductase (NQO1) and heme oxygenase-1 (HO-1), the downstream antioxidant genes of Nrf2 [6] (Figure 4(b)). These results demonstrate that Dox treatment activates Nrf2 in the heart, thus supporting the notion that Nrf2 activation serves as a feedback mechanism to suppress Dox-induced cardiomyopathy by suppression of oxidative stress and enhancement of autophagic clearance of toxic protein aggregates.

### 2.4. Nrf2 Deficiency Exaggerates Dox-Induced Myocardial Oxidative Stress and Impaired Autophagy as well as Cardiac Toxicity and Dysfunction

**Figure 5:**
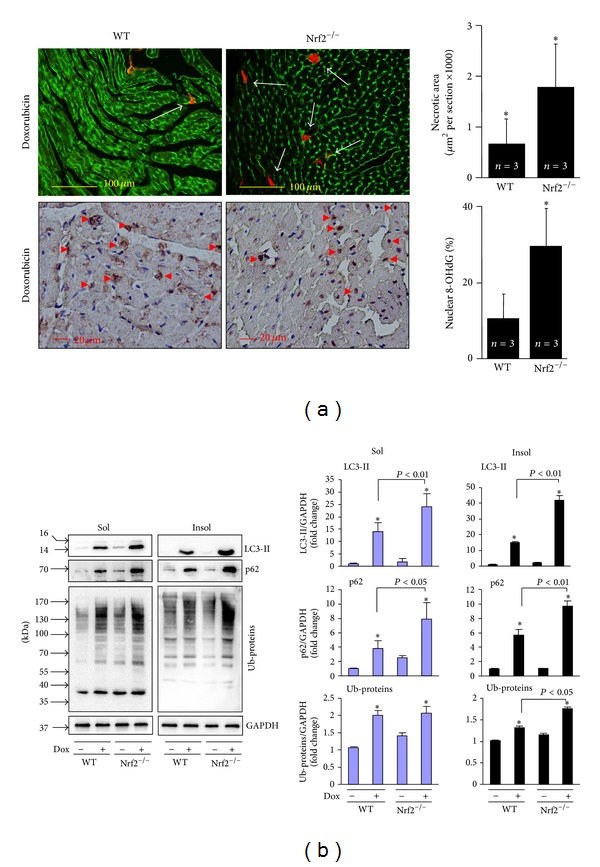
Effects of Nrf2 knockout on Dox-induced myocardial necrosis, oxidative stress, and autophagic clearance. Male 10–12 wk old littermates of WT and Nrf2^−/−^ mice were treated with Dox as in Figure 1 for 4 days and then the hearts (*n* = 3 for each group) were harvested for (a) Evans blue and 8-OHdG staining and (b) Western blot analysis of LC3, p62, and polyubiquitinated proteins (Ub-proteins) in soluble and insoluble fractions. *P < 0.05 versus WT control in (a); *P < 0.05 versus Dox (−) control in the same experimental group.

To explore the pathophysiological significance of Dox-induced Nrf2 activation in the heart, we determined the impact of Nrf2 deficiency on Dox-induced cardiac toxicity and dysfunction with a focus on oxidative stress and autophagic activity in the heart using littermates of WT and Nrf2^−/−^ mice. There was no detectable Evans blue uptake or 8-OHdG staining in the heart of WT and Nrf2^−/−^ mice treated with vehicle (data not shown). As shown in Figure 5 and Table 1, knockout of Nrf2 exaggerated myocardial oxidative stress and necrosis, as well as cardiac dysfunction. To further determine whether Nrf2 regulates autophagic clearance of toxic ubiquitinated protein in Dox-intoxicated heart, we examined the levels of LC3-I and -II, p62, and ubiquitinated proteins in both soluble and insoluble fractions of the hearts from WT and Nrf2^−/−^ mice treated with or without Dox. Loss of Nrf2 enhanced Dox-induced upregulation of LC3-II and p62 proteins in both soluble and insoluble fractions (Figure 5(b)), suggesting that Nrf2 negatively regulates Dox-impaired autophagic flux. Importantly, Nrf2 deficiency did not affect Dox-induced accumulation of ubiquitinated proteins in soluble fractions, while enhancing the accumulation of ubiquitinated proteins in insoluble fractions (Figure 5(b)). Because the toxic ubiquitinated protein aggregates are usually insoluble and predominantly cleared by autophagosome fusion with lysosome, the enhancement of Dox-increased levels of LC3-II, p62, and ubiquitinated proteins in the insoluble fraction of Nrf2^−/−^ mice is most likely due to an impaired autophagic clearance of toxic insoluble ubiquitinated protein aggregates in the Nrf2^−/−^ heart. Taken together, loss of Nrf2 exaggerates Dox-impaired autophagosome clearance thereby leading to the accumulated ubiquitinated proteins in the heart. These results reveal that Nrf2 is likely a critical suppressor of Dox-induced cardiomyopathy via its abilities to inhibit both oxidative stress and insufficient autophagic activity in the heart.

### 2.5. Nrf2 Activation Ameliorates Dox-Induced Autophagy Impairment and Cell Death in Cardiomyocytes via Facilitating Autophagic Clearance of Ubiquitinated Protein Aggregates

**Figure 6:**
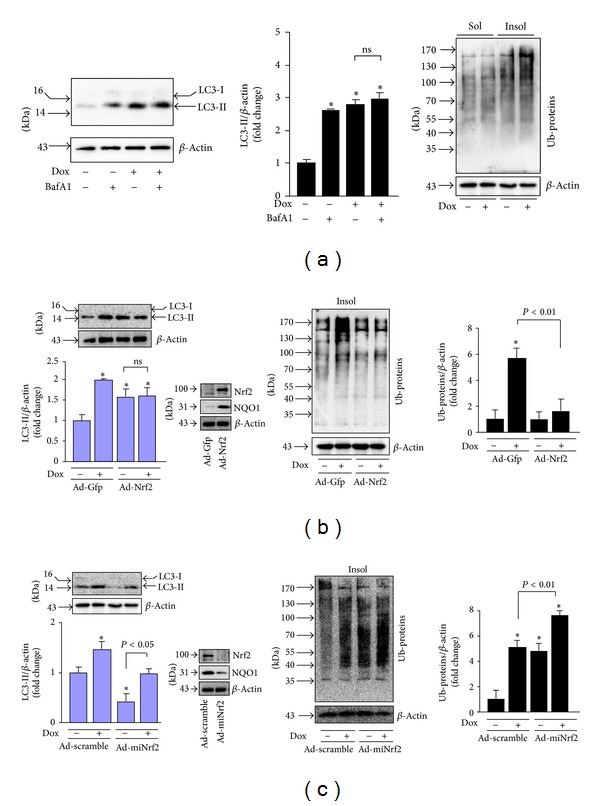
Effects of Nrf2 overexpression and knockdown on Dox-induced impairment of autophagy in cardiomyocytes. (a) Dox-induced impairment of autophagic flux. Rat neonatal cardiomyocytes were treated with Dox (1 *μ*M) in serum-free DMEM for 24 h and bafilomycin A1 (BafA1, 5 nM) was added during the last 4 h. Left panel: representative immunoblots of Dox-induced LC3-II expression with or without BafA1 treatment. Middle panel: quantified densitometric analysis of Dox-induced LC3-II expression. *n* = 4, *P < 0.05 versus vehicle control (−). Right panel: representatives of immunoblots of Dox-induced accumulation of ubiquitinated proteins in soluble (Sol) and insoluble (Insol) fractions. Cells were treated with or without Dox (1 *μ*M) for 24 h. (b) Overexpression of Nrf2 on Dox-induced LC3-II expression and accumulation of ubiquitinated proteins in rat neonatal cardiomyocytes. Infected cells were subjected to Western blot analysis of LC3 and insoluble (Insol) ubiquitinated protein levels. Cells were treated with Dox (1 *μ*M) for 24 h. *n* = 4, *P < 0.05 versus Ad-Gfp (−). Inserted blots: confirming the infection efficiency of Ad-Nrf2. (c) Overexpression of miNrf2 on Dox-induced LC3-II expression and accumulation of ubiquitinated proteins in rat neonatal cardiomyocytes. Infected cells were subjected to Western blot analysis of LC3 and insoluble (Insol) ubiquitinated protein levels. Cells were treated with Dox (1 *μ*M) for 24 h. *n* = 4, *P < 0.05 versus Ad-Scramble (−). Inserted blots: confirming the infection efficiency of Ad-Nrf2.

**Figure 7:**
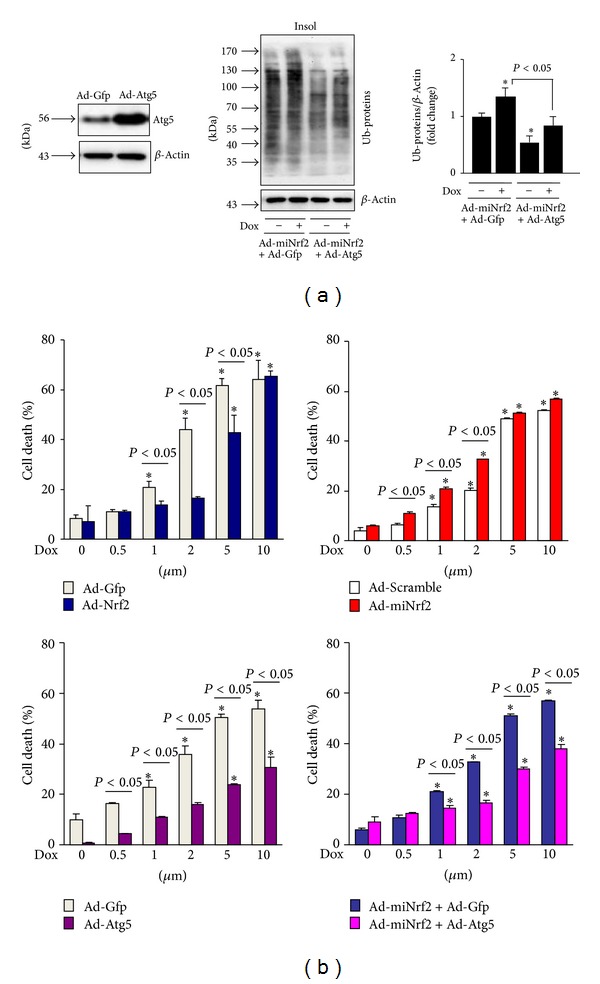
Effects of Nrf2, miNrf2, and Atg5 on Dox-induced cell death in cardiomyocytes. (a), Overexpression of Atg5 on Dox-induced accumulation of ubiquitinated proteins in Nrf2 depleted rat neonatal cardiomyocytes. Cells infected with mNrf2 were further infected with Ad-Gfp or Ad-Atg5 and then treated with Dox (1 *μ*M) for 24 h. *n* = 4, *P < 0.05 versus Ad-miNrf2 + Ad-Gfp (−). (b) Rat neonatal cardiomyocytes infected with Ad-Gfp, Nrf2, Atgr5, Ad-scramble, and Ad-miNrf2 as indicated were treated with or without Dox (1 *μ*M) for 24 h and cell death was assessed by LDH cell death assay kit. *n* = 4, *P < 0.05 versus Dox (0 *μ*M) in the same experimental group.

To further establish a functional link between Nrf2 activation, autophagic clearance of ubiquitinated protein aggregates, and cardiomyocyte survival in a setting of Dox-induced cardiotoxicity, we applied adenoviral Nrf2 and autophagy related gene (Atg)5 gain- and loss-of-function approaches in primary culture of rat neonatal cardiomyocytes. We first determined whether Dox induces autophagic impairment in primary culture of rat neonatal cardiomyocytes. As shown in Figure 6(a), Dox at toxic dose of 1 *μ*M (Figure 7) increased the steady level of LC3-II; however, treatment of bafilomycin A1 (BafA1), an inhibitor of autophagosome fusion with lysosome, did not further accumulate the Dox-induced upregulation of LC3. In addition, the Dox (1 *μ*M) treatment led to accumulation of ubiquitinated proteins in insoluble cellular fractions (Figure 6(a)). These results revealed that Dox-induced cytotoxicity is associated with impaired autophagic activity and accumulated ubiquitinated protein aggregates in cardiomyocytes as observed in the heart after Dox treatment (Figure 3). We then examined whether Nrf2 is capable of regulating the Dox-induced impairment in cultured rat neonatal cardiomyocytes. Overexpression of Nrf2 upregulated whereas Nrf2 knockdown downregulated the steady levels of LC3-II (Figures 6(b) and 6(c)), indicating a potential role of Nrf2 in either increasing autophagosome formation or decreasing autophagosome clearance. Dox treatment accumulated the steady levels of LC3-II in the Ad-Gfp control but not in Ad-Nrf2 infected cells (Figure 6(b)), suggesting a protective role of Nrf2 in Dox-induced impairment of autophagic activation. In contrast, Dox accumulated the steady levels of LC3-II in both Ad-scramble control and Ad-miNrf2 infected cells with a similar magnitude (Figure 6(c)). Importantly, Nrf2 overexpression dramatically inhibited Dox-induced accumulation of ubiquitinated protein aggregates; however, Nrf2 knockdown enhanced basal and Dox-induced accumulation of ubiquitinated protein aggregates (Figures 6(b) and 6(c)). Collectively, these results suggest that Nrf2 plays a critical role in suppressing Dox-induced accumulation of ubiquitinated protein aggregates most likely via enhancing autophagosome formation and autophagic clearance of ubiquitinated protein aggregates in cardiomyocytes. Finally, we investigated the functional significance of Nrf2-mediated enhancement of autophagic clearance of ubiquitinated protein aggregates in a setting of Dox-induced cardiotoxicity in cultured rat neonatal cardiomyocytes. As shown in Figure 7(a), the accumulation of ubiquitinated protein aggregates in Nrf2 depleted cells at basal and Dox treatment conditions was significantly attenuated by forced activation of autophagy via adenoviral overexpression of Atg5, supporting a critical role of autophagy activation for Nrf2-mediated clearance of ubiquitinated protein aggregates in cardiomyocytes. Moreover, Dox-induced cardiomyocyte death was inhibited or enhanced by Nrf2 overexpression or knockdown, respectively (Figure 7(b)). Overexpression of Atg5 potently suppressed cell death in both Nrf2 intact and depleted cardiomyocytes; however, the magnitude of cell death suppression is less in Nrf2 depleted cells (Figure 7(b)), indicating that autophagy activation may be a downstream event in Nrf2-mediated clearance of ubiquitinated protein aggregates in cardiomyocytes. Overall, these findings support the notion that Nrf2 is an endogenous suppressor in Dox-induced cardiomyopathy most likely via facilitating autophagic clearance of myocardial toxic protein aggregates.

## 3. Discussion

In the present study, we have demonstrated for the first time that Nrf2 is a critical inhibitor of Dox-induced cardiac toxicity and dysfunction. Mechanistically, it is likely that, in addition to the suppression of oxidative stress, Nrf2 is capable of activating sufficient myocardial autophagy to prevent Dox-induced cardiomyopathy. These findings not only provide clarification to the controversial observations regarding the role of autophagy in Dox-induced cardiomyopathy but also highlight the potential of targeting Nrf2 as a novel therapeutic strategy to attenuate the Dox-induced cardiotoxicity and cardiomyopathy.

### 3.1. Dox-Impaired Autophagy in the Heart

The previous observations regarding the role of autophagy in Dox-induced cardiotoxicity are controversial [20–22]. In a subacute model of Dox-induced cardiomyopathy in rats, treatment of 3-methyladenine (3-MA), an inhibitor of autophagy activation, attenuates Dox-induced cardiotoxicity and cardiac dysfunction [21]. However, in an acute model of Dox-induced cardiomyopathy in mice, either starvation or treatment with rapamycin, an activator of autophagy, suppresses these adverse events [20, 22]. In addition, Dox has been shown to suppress myocardial autophagic flux [20], while Dox-induced increases in autophagic flux in cultured cardiomyocytes have also been documented [22, 23]. The precise reasons for these discrepancies are unclear. However, the effects of chemical modulators of autophagy* in vivo* need to be interpreted with caution. For example, 3-MA has been widely used as an autophagy inhibitor based on its inhibitory effect on class III phosphoinositide 3-kinase (PI3K) activity, which is known to be essential for the induction of autophagy [24, 25]. Notably, it has also been demonstrated that 3-MA promotes autophagy in nutrient-rich conditions while it suppresses starvation-induced autophagy [26]. Thus, the observed cardioprotective effect of 3-MA treatment in a rat model of Dox-induced cardiotoxicity is most likely due to the activating rather than inhibiting autophagy in the heart [21]. On the other hand, the increases in autophagic activities may result in either protective or detrimental consequences in the heart [27, 28]. The precise mechanisms underlying are unknown. A plausible explanation is likely that insufficient activation of myocardial autophagy, that is, increased autophagosome formation without sufficient fusion with lysosomes and/or the subsequent lysosome-mediated clearance of autophagosomes, is functionally impaired and detrimental to the heart; whereas sufficient activation of myocardial autophagy, that is, increased autophagosome formation with sufficient fusion with lysosomes and the subsequent lysosome-mediated clearance of autophagosomes, is functionally integrated and therefore cardioprotective. Taken together, these results suggest that Dox most likely induces insufficient activation of autophagy in the heart and myocardial sufficient activation of autophagy could attenuate Dox-induced cardiotoxicity. In the present study, we further linked the impaired autophagy to Dox-induced cardiotoxicity and cardiac dysfunction, by demonstrating that Dox treatment led to the accumulation of LC3-II, p62, and ubiquitinated proteins, impaired myocardial autophagy flux, and minor alternations of lysosomal function, as well as cardiomyocyte necrosis and cardiac dysfunction. In cultured cardiomyocytes, we further demonstrated that Dox treatment resulted in impairment of autophagic flux, accumulation of ubiquitinated protein aggregates, and cell death. Moreover, Dox-induced accumulation of ubiquitinated protein aggregates and cell death in cardiomyocytes were inhibited by forced activation of autophagy via adenoviral overexpression of Atg5. These results reveal that Dox treatment instead of activating autophagy activity in the heart actually suppresses it thereby contributing to cardiotoxicity and cardiac dysfunction. The observed Dox-induced upregulation of LC3-II steady levels in the heart is likely due to the impairment of autophagosome clearance rather than the activation of autophagosome formation.

It is worthy to note that, while Dox may transiently activate the UPS system in cardiomyocytes [29], the chronic and cumulative effect of Dox actually suppresses UPS function resulting in the accumulation of damaged and toxic ubiquitinated proteins in the heart [23, 30]. Autophagy is activated in cultured cardiomyocytes by relative long-term treatments (>18 h) of Dox when it is capable of causing accumulation of polyubiquitinated proteins [22, 23, 31]. It is unclear whether the observed activation of autophagy in cardiomyocytes* in vitro* is secondary to the Dox-induced suppression of UPS function, or instead Dox is able to activate autophagy in cultured cardiomyocytes as a result of the specific experimental conditions. However, our results and those of others have demonstrated that Dox treatment could impair autophagic activity and accumulation of ubiquitinated protein aggregates in cardiomyocytes and in the heart. Importantly, when UPS is functionally impaired, the ubiquitinated soluble abnormal proteins accumulate and aggregate into insoluble and toxic protein aggregates. As a result autophagy is activated by default and becomes the major clearance route of the ubiquitinated proteins [17, 18]. Given the Dox-induced functional insufficiency of UPS in the heart, the loss of sufficient autophagic activity may be a major cause of Dox-induced cardiotoxicity.

### 3.2. An Inhibitory Role of Nrf2 in Dox-Mediated Impairment of Myocardial Autophagy

Considering the aforementioned critical role of Nrf2 in suppressing oxidative stress and facilitating autophagic clearance of toxic ubiquitinated proteins in the heart, we were not surprised to observe the exaggerated cardiac necrosis and dysfunction as well as the increased oxidative stress and enhanced impairment of autophagic clearance in Dox-intoxicated hearts due to the loss of Nrf2 function. Intriguingly, it was unexpected that Dox-induced cardiac oxidative stress is modestly enhanced by knockout of Nrf2. Thus, these results further support our notion that Nrf2 is able to sufficiently activate autophagy via a mechanism independent of its ability to suppress oxidative stress. Indeed, we demonstrated that Nrf2 plays a critical mediator role in suppressing Dox-induced autophagy impairment and cell death in cultured cardiomyocytes most likely via facilitating autophagic clearance of toxic ubiquitinated protein aggregates, which is independent of ROS formation.

There are sophisticated interactions between oxidative stress and autophagy in the heart [32]. Given a mediator role of ROS in the induction of autophagy, the overproduced ROS could activate autophagy to serve as a predominantly prosurvival mechanism in a setting of oxidative stress; however, the autophagy machinery might also be self-destructive when it is induced insufficiently thereby exaggerating oxidative stress. Indeed, ROS-mediated autophagy has been shown to lead to either survival or death in cardiac cells depending on stimuli and cell types [33–35]. Thus it is likely that ROS may induce either sufficient or insufficient activation of myocardial autophagy thereby protecting against or leading to, respectively, cardiac dysfunction. The precise mechanisms for these opposite effects are unknown. Nevertheless, the observations that Nrf2 deficiency exaggerated both oxidative stress and impairment of autophagic clearance in Dox-intoxicated hearts and Nrf2 plays a mediator role in suppressing Dox-induced autophagy impairment and cell death in cultured cardiomyocytes suggest that Nrf2 plays a critical role in the regulation of myocardial activation of sufficient and frustrated autophagy in the setting of oxidative stress.

### 3.3. Conclusion

Nrf2 is likely an endogenous inhibitor of Dox-induced cardiomyopathy. Mechanistically, Nrf2 is capable of suppressing Dox-induced oxidative stress and impaired autophagy. Further investigation of molecular mechanisms by which Nrf2 regulates the interplay between oxidative stress and insufficient autophagy in Dox-induced cardiotoxicity will provide valuable insights into the feasibility of targeting Nrf2 as a novel strategy for preventing or mitigating Dox-induced cardiotoxicity and cardiac dysfunction.

## 4. Materials and Methods

### 4.1. Animals

Male C57BL/6J mice were purchased from Shanghai Slac Laboratory Animal Co., Ltd. Breeding pairs of heterozygous Nrf2 knockout (Nrf2^+/−^/C57BL/6J) mice were purchased from Riken BioRosource Center, Japan, and housed under standard conditions in the Institution's AAALAC approved animal facility. Littermates of wild type (WT; Nrf2^+/+^) and homozygous Nrf2 knockout (Nrf2^−/−^) mice were generated using the Nrf2^+/−^ breeding pairs as previously described [36]. Mice were euthanized by intraperitoneal (IP) injection of pentobarbital sodium (100 mg/kg). All of the animal protocols were conducted in accordance with the NIH* Guide for Care and Use of Laboratory Animals* and were approved by the Institutional Animal Care and Use Committee at Shandong University, China, and the University of South Carolina, USA.

### 4.2. Doxorubicin-Induced Cardiomyopathy

Male 10–12 wk old mice were subjected to single intraperitoneal injections of either 25 mg/kg doxorubicin HCl (Dox) (Aladdin Reagent Inc., China) dissolved in 0.9% NaCl or 0.9% NaCl vehicle as previously reported [12].

### 4.3. Echocardiography

Echocardiography was performed on anesthetized (1.5% isoflurane) mice using the Vevo 770 High-Resolution Imaging System (VisualSonics Inc.) with a 35-MHz high-frequency linear transducer as previously described [6]. Left ventricle (LV) internal dimension (LVID; in mm), intraventricular septal thickness (IVS; in mm), and LV posterior wall thickness (LVPW; in mm) were measured. LV percent fractional shortening FS (%) was calculated via VisualSonics Measurement Software.

### 4.4. Measurement of Autophagic Flux* In Vivo*

Autophagic flux* in vivo* was measured as previously described with a minor modification [37]. Briefly, mice were intraperitoneally injected with rapamycin (2 mg/kg) or rapamycin (2 mg/kg) plus chloroquine (10 mg/kg) 4 hrs prior to harvesting hearts. LV tissue lysates were subjected to Western blot analysis of LC3-I and -II. The chloroquine-induced accumulation of LC3-II was considered to be autophagic flux.

### 4.5. Histology and Immunochemistry

Hearts were cannulated via the LV apex, cleared by perfusion with 0.9% NaCl at 90 mmHg, arrested in diastole with 60 mM KCl, fixed by perfusion with 10% formalin, and embedded in paraffin. Paraffin sections were prepared (5 *μ*m, Leica, rotary microtome) and stored at room temperature until staining. Hematoxylin-Eosin (HE) staining and immunohistochemistry were performed as previously described [6]. Nrf2 immunohistochemistry was performed using a rabbit anti-mouse Nrf2 polyclonal antibody (C-20, Santa Cruz Biotechnology, Inc.). Staining of 8-hydroxydeoxyguanosine (8-OHdG), a marker of DNA oxidization in the heart, was performed using a mouse anti-8-OHdG antibody (sc-660369, Santa Cruz Biotechnology, Inc.).

### 4.6. Evans Blue Labelling

Evans blue dye becomes insanely red fluorescent when conjugated to albumin in the circulation. The dye/albumin complexes are excluded from cells with intact plasma membranes while accumulating in damaged myofibers when the muscle cell membrane is broken, thus providing a dye-exclusion viability test. The red auto-fluorescence accumulated in myocardium has been used as a histopathological sign of cardiomyocyte necrosis [14]. Briefly, mice were subjected to a single intraperitoneal injection of Evans blue (100 mg/kg, Solarbio science & technology Co., Ltd., Beijing, China) 18 h prior to harvesting tissues. Harvested hearts were fixed in 4% paraformaldehyde and then embedded in paraffin. Paraffin sections were prepared (5 *μ*m, Leica, rotary microtome) and stored at room temperature until staining. Myocardial cellular membranes were stained with Wheat Germ Agglutinin, Alexa Fluor 488 Conjugate (Invitrogen Corp.). Sections were observed under fluorescence microscope (Leica Microsystems Ltd.) at 200x magnification. Evans blue dye-positive area (red) indicates cardiomyocyte necrosis.

### 4.7. Virus Preparation

Adenovirus of miNrf2 (Ad-miNrf2) was generated using BLOCK-iT Pol II miR RNAi Expression Vector Kits (Gateway-adapted expression vector for the expression of microRNA (miRNA) in mammalian cells under control of Pol II promoters) (Invitrogen, K4936-00) as well as ViraPower Adenoviral Promoterless Gateway Expression Kit (Invitrogen, K4940-00). Briefly, double-stranded rat Nrf2 miRNA (miNrf2) (5′-TACACAGGGACAGATCACAGC-3′) was cloned into pcDNA 6.2-GW/EmGFP-miR expression vector using T4 DNA ligase. After a BP recombination (Invitrogen, 11789020) reaction with pcDNA 6.2-GW/EmGFP-miR and pDONR 221 (Invitrogen, 12536017) vector to get a miR RNAi entry vector, LR recombination (Invitrogen, 12538120) reaction with entry vector and pAd/CMV/V5-DEST vector was performed to generate a pAd-miNrf2 vector. Transfection of the expression clone into 293A cells was performed to produce Ad-miNrf2 stock. Adenovirus of miScramble was generated using pcDNA 6.2-GW/EmGFP-miR-neg control plasmid which is provided in the BLOCK-iT Pol II miR RNAi Expression Vector Kits. Ad-miScramble stock was generated as described above.

Adenovirus of Nrf2 (Ad-Nrf2) and Atg5 (Ad-Atg5) was generated using pENTR 1A Dual Selection Vector (Invitrogen, A10462) and ViraPower Adenoviral Promoterless Gateway Expression Kit (Invitrogen, K4940-00). Briefly, human Atg5 cDNA (pCMV-myc-Atg5, addgen 24922) and human Nrf2 cDNA (NCBI Reference Sequence: NM_006164.4) were cloned into a Topo blunt zero vector, respectively, to generate a Tope-Atg5 and a Topo-Nrf2 vector. Then the Atg5 and Nrf2 coding sequences were reconstructed into the pENTR1A-GFP vector to form pENTR1A-GFP-Atg5 and pENTR1A-GFP-Nrf2 vectors. LR recombination (Invitrogen, 12538120) reaction with the entry vector of pENTR1A-GFP-Atg5 and pENTR1A-GFP-Nrf2 with pAd/CMV/V5-DEST vector was performed to generate pAd-Atg5 and pAd-Nrf2 vectors. Adenovirus of Gfp (Ad-Gfp) was generated using the pENTER1A-Gfp vector as described above. Transfection of these expression vectors into 293A cells to produce the adenoviral stock.

### 4.8. Cell Culture and Adenovirus Infection

Rat neonatal cardiac myocytes were isolated and cultured as previously described [6]. Briefly, the hearts from 3-day old Wistar rats were finely minced and digested with type II collagenase (120 units/mL; Worthington Biochemical Corp., Lakewood, NJ). Dispersed cells were placed in culture flask for 80 minutes at 37°C in a CO_2_ incubator. During this time, only the fibroblasts became attached to the culture flask. The purified cardiomyocytes, the viability of which was >85% determined by trypan blue exclusion, were seeded onto gelatin-coated plastic culture dishes at a density of 5 × 10^4^ cells/cm^2^ in low glucose DMEM supplemented with 8% horse serum, 5% new-born calf serum, penicillin (100 U/mL), and streptomycin (100 mg/mL). Nrf2 gain or loss-of-function approaches were achieved by infecting the cells with adenovirus of GFP, Nrf2, scramble microRNA, or rat Nrf2 microRNA in serum free DMEM for 8 hours and then changed to DMEM supplemented with 1.6% horse serum and 1% new-born calf serum for totally 48 hours. Ad-Atg5 infection was performed as described above.

Adenoviral overexpression of Nrf2 (20 MOI) or Nrf2 microRNA (20 MOI) resulted in dramatic Nrf2 and downstream NQO1 protein expression or up to 80% knockdown of Nrf2 and downstream NQO1 protein expression in rat neonatal cardiomyocytes, without any apparent cytotoxic effects (data not shown). Ad-Atg5 (20 MOI) infection sufficiently led to increased levels of ATG5 in rat neonatal cardiomyocytes. Accordingly, we used adenoviral overexpression of Nrf2, Nrf2 microRNA, and Atg5 at a dose of 20 MOI in rat neonatal cardiomyocytes.

Cell death was measured using a cytotoxicity detection kit (Roche, 04 744 934 001). Cell viability was calculated as follows: cell viability = (LDH activity of cell lysate in Exp.)/(LDH activity of cell lysate in Con.) × 100%. Exp. indicates experimental groups and Con. is for control.

### 4.9. Western Blot Analysis

Western blot analysis was performed as previously described [6]. Regular Western blot analysis was performed using LV lysates that were prepared using RIPA buffer (50 mM Tris (pH 8.0), 150 mM NaCl, 1% NP-40, 0.5% sodium deoxycholate, 0.1% SDS, and a protein inhibitor mixture (Sigma-Aldrich)). In addition, Western blot analysis of protein aggregation was performed using LV detergent-soluble and -insoluble lysates. Briefly, detergent-soluble fractions of LV tissues were obtained using a 2% Triton X-100 lysis buffer (50 mM Tris (pH 8.0), 150 mM NaCl, 1 mM EDTA, 10% glycerol, 2% Triton X-100, and a protein inhibitor mixture (Sigma-Aldrich)). The insoluble fractions were further solubilized using the 2% Triton X-100 lysis buffer supplemented with 1% SDS. Primary antibodies included anti-LC3 (L7543, Sigma-Aldrich), anti-P62 (ab91526, abcam), anti-NQO1 (sc-376023, Santa Cruz Biotechnology, Inc.), Anti-ATG5 (A2859, Sigma-Aldrich), anti-ATG7 (A2856, Sigma-Aldrich), anti-LAMP1 (ab24170, Abcam), anticathepsin D (2284, CST), and anti-Ub (sc-8017, Santa Cruz Biotechnology, Inc.). Ubiquitinated proteins with molecular weights from the top to 35 kDa on the membranes of immunoblots were quantified

### 4.10. Reverse Transcription-Polymerase Chain Reaction (RT-PCR) and Quantitative Real Time (Q-PCR)

Total RNA from the LV was extracted using RNeasy Fibrous Tissue Mini kit (Qiagen Inc., Valencia, CA), and reverse transcription reactions (SuperScript III First-Strand Synthesis System for RT-PCR, Invitrogen Corp., Carlsbad, CA) were performed with 0.5 *μ*g of DNase I (Qiagen)-treated RNA. PCR and quantitative real-time PCR (Q-PCR) were carried out using the CFX96 Touch Real-Time PCR Detection System (Bio-Rad, Laboratories, Inc.). Expression levels of target genes were normalized by concurrent measurement of glyceraldehyde-3-phosphate dehydrogenase (GAPDH) mRNA levels as previously described [6]. Primers that were used for Q-PCR are summarized in Table 2.

### 4.11. Statistical Analysis

Data are shown as mean ± SD. Statistical differences were analyzed by one-way ANOVA followed by Bonferroni test for multiple comparisons using GraphPad Prism software. Differences were considered significant at *P* < 0.05.

## Figures and Tables

**Table 1:** Time-course study of echocardiography in WT and Nrf2^−/−^ mice after Dox treatment.

	WT		Nrf2^−/−^	
	(*n*) Vehicle (3)	Dox (5)	Vehicle (3)	Dox (5)
Day 0				
LVPW; d (mm)	0.69 ± 0.02	0.65 ± 0.05	0.67 ± 0.04	0.68 ± 0.03
LVPW; s (mm)	1.05 ± 0.02	1.05 ± 0.05	0.98 ± 0.04	0.98 ± 0.03
FS (%)	28.90 ± 1.56	28.31 ± 3.97	29.50 ± 2.00	28.53 ± 1.19
LV Vol; d (µL)	60.41 ± 0.02	60.86 ± 0.01	61.56 ± 0.07	60.53 ± 0.02
LV Vol; s (µL)	23.68 ± 0.04	23.78 ± 0.01	24.08 ± 0.07	23.81 ± 0.03
HR	469.27 ± 52.42	423.62 ± 29.97	438.00 ± 38.73	426.70 ± 50.89
Day 1				
LVPW; d (mm)	0.69 ± 0.03	0.68 ± 0.02	0.66 ± 0.01	0.65 ± 0.03
LVPW; s (mm)	1.07 ± 0.03	1.01 ± 0.02	0.99 ± 0.01	0.90 ± 0.03^B;C^
FS (%)	31.12 ± 2.90	28.23 ± 1.10	31.03 ± 2.00	20.98 ± 1.94^B;C^
LV Vol; d (µL)	61.78 ± 0.09	61.92 ± 0.03	63.02 ± 0.01	60.45 ± 0.02
LV Vol; s (µL)	24.40 ± 0.08	24.10 ± 0.03	24.92 ± 0.07	22.91 ± 0.03
HR	442.06 ± 16.11	429.90 ± 20.29	439.22 ± 11.07	431.73 ± 32.97
Day 2				
LVPW; d (mm)	0.71 ± 0.09	0.67 ± 0.05	0.69 ± 0.06	0.60 ± 0.02
LVPW; s (mm)	1.07 ± 0.09	0.97 ± 0.05 ^A^	0.97 ± 0.06	0.83 ± 0.02^B;C^
FS (%)	28.03 ± 1.05	23.00 ± 3.37^A^	30.29 ± 6.02	17.89 ± 3.16^B;C^
LV Vol; d (µL)	62.47 ± 0.04	60.82 ± 0.01	60.92 ± 0.02	58.73 ± 0.07^B;C^
LV Vol; s (µL)	25.80 ± 0.03	24.01 ± 0.02	24.35 ± 0.02	20.99 ± 0.02^B;C^
HR	451.67 ± 26.50	445.38 ± 20.20	452.44 ± 28.66	444.73 ± 20.33
Day 4				
LVPW; d (mm)	0.70 ± 0.06	0.67 ± 0.02	0.66 ± 0.01	0.59 ± 0.03^B;C^
LVPW; s (mm)	1.08 ± 0.06	0.96 ± 0.02^A^	0.97 ± 0.01	0.79 ± 0.03^B;C^
FS (%)	28.60 ± 0.80	21.71 ± 3.40 ^A^	29.13 ± 1.40	18.49 ± 1.36^B;C^
LV Vol; d (µL)	63.17 ± 0.04	58.88 ± 0.08^A^	60.39 ± 0.03	57.51 ± 0.03^B;C^
LV Vol; s (µL)	24.28 ± 0.01	20.21 ± 0.01^A^	22.96 ± 0.02	19.34 ± 0.08^B;C^
HR	444.06 ± 13.36	437.33 ± 29.66	443.89 ± 19.76	427.60 ± 29.83

Male 10–12 wk old littermates of WT and Nrf2^−/−^ mice were treated with single intraperitoneal injection of 25 mg/kg Dox or vehicle (Veh) control for 4 days and cardiac function was monitored daily as indicated. LVPW; d: left ventricular posterior wall thickness diastolic; LVPW; s: left ventricular posterior wall thickness systolic; FS: fractional shortening; LV Vol; d: left ventricular volume; diastolic; LV Vol; s: left ventricular volume; systolic; HR: heart rate. Animal number for each group is indicated in the parentheses. ^A^ *P* < 0.05 versus WT Vehicle; ^B^ *P* < 0.05 versus Nrf2^−/−^ Vehicle; ^C^ *P* < 0.05 versus WT DOX.

**Table 2:** Primers for real-time PCR.

Genes	Gene access #	Forward	Reverse	Product length (bp)
Nrf2 NQO-1 HO-1 GAPDH	NM_010902.3 NM_008706.5 NM_010442.2 XM_001479322	ATGATGGACTTGGAGTTGCC CGGTATTACGATCCTCCCTCAACA AGGAGATAGAGCGCAACAAGCAGA ATGTTCCAGTATGACTCCACTCACG	TCCTGTTCCTTCTGGAGTTG AGCCTCTACAGCAGCCTCCTTCAT CCAGTGAGGCCCATACCAGAAG GAAGACACCAGTAGACTCCACGACA	200 120 116 171

## References

[1] Singal PK, Iliskovic N (1998). Doxorubicin-induced cardiomyopathys. *The New England Journal of Medicine*.

[2] Volkova M, Russell R Anthracycline cardiotoxicity: prevalence, pathogenesis and treatment. *Current Cardiology Reviews*.

[3] Sterba M, Popelova O, Vavrova A Oxidative stress, redox signaling, and metal chelation in anthracycline cardiotoxicity and pharmacological cardioprotection. *Antioxidants & Redox Signaling*.

[4] Octavia Y, Tocchetti CG, Gabrielson KL, Janssens S, Crijns HJ, Moens AL (2012). Doxorubicin-induced cardiomyopathy: from molecular mechanisms to therapeutic strategies. *Journal of Molecular and Cellular Cardiology*.

[5] Li J, Ichikawa T, Janicki JS, Cui T (2009). Targeting the Nrf2 pathway against cardiovascular disease. *Expert Opinion on Therapeutic Targets*.

[6] Li J, Ichikawa T, Villacorta L (2009). Nrf2 protects against maladaptive cardiac responses to hemodynamic stress. *Arteriosclerosis, Thrombosis, and Vascular Biology*.

[7] Li J, Zhang C, Xing Y (2011). Up-regulation of p27kip1 contributes to Nrf2-mediated protection against angiotensin II-induced cardiac hypertrophy. *Cardiovascular Research*.

[8] Calvert JW, Elston M, Nicholson CK (2010). Genetic and pharmacologic hydrogen sulfide therapy attenuates ischemia-induced heart failure in mice. *Circulation*.

[9] Sussan TE, Rangasamy T, Blake DJ (2009). Targeting Nrf2 with the triterpenoid CDDO-imidazolide attenuates cigarette smoke-induced emphysema and cardiac dysfunction in mice. *Proceedings of the National Academy of Sciences of the United States of America*.

[10] Xing Y, Niu T, Wang W Triterpenoid dihydro-CDDO-trifluoroethyl amide protects against maladaptive cardiac remodeling and dysfunction in mice: a critical role of Nrf2. *PLoS ONE*.

[11] Fujita K-I, Maeda D, Xiao Q, Srinivasula SM (2011). Nrf2-mediated induction of p62 controls Toll-like receptor-4-driven aggresome-like induced structure formation and autophagic degradation. *Proceedings of the National Academy of Sciences of the United States of America*.

[12] Kizaki K, Ito R, Okada M (2006). Enhanced gene expression of myocardial matrix metalloproteinases 2 and 9 after acute treatment with doxorubicin in mice. *Pharmacological Research*.

[13] Zhang Y, Kang Y-M, Tian C (2011). Overexpression of Nrdp1 in the heart exacerbates doxorubicin-induced cardiac dysfunction in mice. *PLoS ONE*.

[14] Miller DL, Li P, Dou C, Armstrong WF, Gordon D (2007). Evans blue staining of cardiomyocytes induced by myocardial contrast echocardiography in rats: evidence for necrosis instead of apoptosis. *Ultrasound in Medicine and Biology*.

[15] Nicholson DW, Ali A, Thornberry NA (1995). Identification and inhibition of the ICE/CED-3 protease necessary for mammalian apoptosis. *Nature*.

[16] Mizushima N, Yoshimori T, Levine B (2010). Methods in mammalian autophagy research. *Cell*.

[17] Rubinsztein DC (2006). The roles of intracellular protein-degradation pathways in neurodegeneration. *Nature*.

[18] Wong E, Cuervo AM (2010). Integration of clearance mechanisms: the proteasome and autophagy. *Cold Spring Harbor Perspectives in Biology*.

[19] Klionsky DJ, Abeliovich H, Agostinis P Guidelines for the use and interpretation of assays for monitoring autophagy in higher eukaryotes. *Autophagy*.

[20] Kawaguchi T, Takemura G, Kanamori H Prior starvation mitigates acute doxorubicin cardiotoxicity through restoration of autophagy in affected cardiomyocytes. *Cardiovascular Research*.

[21] Lu L, Wu W, Yan J, Li X, Yu H, Yu X (2009). Adriamycin-induced autophagic cardiomyocyte death plays a pathogenic role in a rat model of heart failure. *International Journal of Cardiology*.

[22] Sishi BJ, Loos B, van Rooyen J, Engelbrecht AM Autophagy upregulation promotes survival and attenuates doxorubicin-induced cardiotoxicity. *Biochemical Pharmacology*.

[23] Dimitrakis P, Romay-Ogando MI, Timolati F, Suter TM, Zuppinger C Effects of doxorubicin cancer therapy on autophagy and the ubiquitin-proteasome system in long-term cultured adult rat cardiomyocytes. *Cell and Tissue Research*.

[24] Seglen PO, Gordon PB (1982). 3-Methyladenine: specific inhibitor of autophagic/lysosomal protein degradation in isolated rat hepatocytes. *Proceedings of the National Academy of Sciences of the United States of America*.

[25] Petiot A, Ogier-Denis E, Blommaart EFC, Meijer AJ, Codogno P (2000). Distinct classes of phosphatidylinositol 3′-kinases are involved in signaling pathways that control macroautophagy in HT-29 cells. *Journal of Biological Chemistry*.

[26] Wu Y-T, Tan H-L, Shui G (2010). Dual role of 3-methyladenine in modulation of autophagy via different temporal patterns of inhibition on class I and III phosphoinositide 3-kinase. *Journal of Biological Chemistry*.

[27] Gottlieb RA, Mentzer RM (2009). Autophagy during cardiac stress: Joys and frustrations of autophagy. *Annual Review of Physiology*.

[28] Wang ZV, Rothermel BA, Hill JA (2010). Autophagy in hypertensive heart disease. *Journal of Biological Chemistry*.

[29] Liu J, Zheng H, Tang M, Ryu Y-C, Wang X (2008). A therapeutic dose of doxorubicin activates ubiquitin-proteasome system-mediated proteolysis by acting on both the ubiquitination apparatus and proteasome. *American Journal of Physiology—Heart and Circulatory Physiology*.

[30] Sishi BJ, Loos B, van Rooyen J, Engelbrecht AM Doxorubicin induces protein ubiquitination and inhibits proteasome activity during cardiotoxicity. *Toxicology*.

[31] Kobayashi S, Volden P, Timm D, Mao K, Xu X, Liang Q (2010). Transcription factor GATA4 inhibits doxorubicin-induced autophagy and cardiomyocyte death. *Journal of Biological Chemistry*.

[32] Gurusamy N, Das DK (2009). Autophagy, redox signaling, and ventricular remodeling. *Antioxidants and Redox Signaling*.

[33] Younce CW, Kolattukudy PE (2010). MCP-1 causes cardiomyoblast death via autophagy resulting from ER stress caused by oxidative stress generated by inducing a novel zinc-finger protein, MCPIP. *Biochemical Journal*.

[34] Yuan H, Perry CN, Huang C (2009). LPS-induced autophagy is mediated by oxidative signaling in cardiomyocytes and is associated with cytoprotection. *American Journal of Physiology—Heart and Circulatory Physiology*.

[35] Marambio P, Toro B, Sanhueza C (2010). Glucose deprivation causes oxidative stress and stimulates aggresome formation and autophagy in cultured cardiac myocytes. *Biochimica et Biophysica Acta—Molecular Basis of Disease*.

[36] Itoh K, Chiba T, Takahashi S (1997). An Nrf2/small Maf heterodimer mediates the induction of phase II detoxifying enzyme genes through antioxidant response elements. *Biochemical and Biophysical Research Communications*.

[37] Iwai-Kanai E, Yuan H, Huang C (2008). A method to measure cardiac autophagic flux *in vivo*. *Autophagy*.

